# A Novel YOLOv10-Based Algorithm for Accurate Steel Surface Defect Detection

**DOI:** 10.3390/s25030769

**Published:** 2025-01-27

**Authors:** Liefa Liao, Chao Song, Shouluan Wu, Jianglong Fu

**Affiliations:** 1 Jiangxi University of Science and Technology, Nanchang 330000, China; liaolf@xjtu.edu.cn (L.L.); 6720231486@mail.jxust.edu.cn (C.S.); 6720231482@mail.jxust.edu.cn (S.W.); 2 Jiangxi Modern Polytechnic College, Nanchang 330000, China; 3 Big Data Technology Innovation Center of Zhangjiakou, Zhangjiakou 075000, China; 4 Hebei University of Architecture, Zhangjiakou 075000, China

**Keywords:** steel plate defect detection, deep learning, YOLOv10n-SFDC model, YOLOv10, surface detection

## Abstract

To address challenges like manual processes, complicated detection methods, high false alarm rates, and frequent errors in identifying defects on steel surfaces, this research presents an innovative detection system, YOLOv10n-SFDC. The study focuses on the complex dependencies between parameters used for defect detection, particularly the interplay between feature extraction, fusion, and bounding box regression, which often leads to inefficiencies in traditional methods. YOLOv10n-SFDC incorporates advanced elements such as the DualConv module, SlimFusionCSP module, and Shape-IoU loss function, improving feature extraction, fusion, and bounding box regression to enhance accuracy. Testing on the NEU-DET dataset shows that YOLOv10n-SFDC achieves a mean average precision (mAP) of 85.5% at an Intersection over Union (IoU) threshold of 0.5, a 6.3 percentage point improvement over the baseline YOLOv10. The system uses only 2.67 million parameters, demonstrating efficiency. It excels in identifying complex defects like ’rolled in scale’ and ’inclusion’. Compared to SSD and Fast R-CNN, YOLOv10n-SFDC outperforms these models in accuracy while maintaining a lightweight architecture. This system excels in automated inspection for industrial environments, offering rapid, precise defect detection. YOLOv10n-SFDC emerges as a reliable solution for the continuous monitoring and quality assurance of steel surfaces, improving the reliability and efficiency of steel manufacturing processes.

## 1. Introduction

Steel is an indispensable material in modern industrial manufacturing, widely utilized in sectors such as aerospace, automotive, and chemical industries [1]. As a structural material, steel directly influences product quality, project safety, and economic benefits. Consequently, the quality control of steel has emerged as a critical aspect of industrial production, with the detection of surface defects being a primary focus [2]. Surface defects in steel not only diminish its mechanical properties but may also pose safety hazards and adversely affect the product’s service life and performance [3]. Therefore, the timely and accurate detection and repair of these surface defects are essential to ensure the quality of steel.

Surface defects in steel, such as cracks and scratches [4], can arise from material quality, improper production practices, or environmental factors, and while minor defects may have minimal impact, most can significantly reduce the steel’s strength and plasticity [5], potentially leading to fractures or failure. Therefore, detecting these defects is crucial for both quality assessment and ensuring safety in engineering applications. Traditional steel surface defect detection technology is primarily categorized into two methods: manual inspection and physical inspection. Manual inspection, one of the earliest techniques, often relies on experienced workers to visually assess the steel’s surface. While this method can swiftly identify some obvious defects, it has significant drawbacks, including low detection efficiency, substantial error margins, and susceptibility to factors such as worker fatigue and emotional state [6]. Furthermore, manual inspection is highly subjective, making it challenging to maintain long-term consistency and high precision, particularly in large-scale production settings with stringent quality control requirements [7].

To address the limitations of manual detection, various physical detection methods have been developed. Among the commonly used methods are eddy current testing, magnetic flux leakage testing, infrared detection, and laser scanning. Eddy current testing [8,9] is effective for detecting surface cracks and corrosion, while magnetic flux leakage testing [10,11] is particularly sensitive when identifying surface defects in magnetic steels. Infrared inspection [12,13] reveals defects by measuring temperature changes on the object’s surface, whereas laser scanning [14] provides a three-dimensional scan of the steel surface to identify minute defects. Although these physical inspection methods offer advantages in enhancing detection precision, they often necessitate costly equipment and impose stringent requirements on the operating environment and inspection conditions, thereby limiting their widespread application in real-world industrial settings. Furthermore, traditional physical testing methods are generally slow, which hampers their ability to meet the demands of efficient, high-volume steel production.

With the rapid advancements in machine vision and deep learning technologies, detecting steel surface defects through image processing has become a focal point of research [15]. Machine vision systems [16] capture images of the steel surface, which are then processed by deep learning models for automated defect detection. This approach enhances detection precision, reduces manual errors, and efficiently handles complex scenarios. This study presents an enhanced defect detection model for steel surfaces based on YOLOv10, focusing on improving detection precision and real-time performance. By optimizing the network architecture and introducing novel feature fusion techniques, the model significantly enhances its ability to detect a wide range of steel surface defects, while reducing computational overhead and ensuring efficient operation in industrial environments.

## 2. Related Work

In recent years, with the continuous development of deep learning technologies, significant progress has been made in the field of steel surface defect detection. Several deep learning-based methods have been proposed to detect steel surface defects [17]. For instance, Lv et al. [18] used the YOLOv8 model with MobileViTv2 and Cross-Local Connection, achieving a 74.1% mAP50. Wang et al. [19] enhanced YOLO-V7 for steel strip defect detection, integrating BiFPN, ECA attention, and SIoU loss for higher precision. Gao et al. [20] developed YOLOv5-KBS, combining attention mechanisms and BiFPN, achieving 79.9% mAP at 70 fps. Similarly, Fityanul Akhyar et al. [21] proposed the FDD system, improving detection precision by incorporating deformable convolutions and ROI pooling. In a different approach, Praveen Damacharla et al. [22] introduced TLU-Net, a transfer learning-based U-Net framework that improves segmentation with limited data. Prashant Kumar Uraon et al. [23] combined FPN and ResNet, achieving a Dice score of 0.7963 and an IoU score of 0.6818. Mingshan Liu et al. [24] used U-Net for feature extraction and defect identification, meeting the needs of automated quality inspection.

YOLO algorithms have been applied to various materials, including metal and stone. In the wood surface defect detection field, Xi et al. [25] proposed SiM-YOLO, achieving a 9.3% mAP increase. Wang et al. [26] proposed CTDD-YOLO, a lightweight model for tile defects, boosting mAP by 7.2% while reducing parameters compared to YOLOv8n. Xu et al. [27] proposed GEB-YOLO, an optimized YOLOv7 model for aluminum profile surface defect detection, improving accuracy by 6.3% and speed by 15% through the integration of GAM, EVCBlock, and BiFPN. Li et al. [28] introduced MSFE-YOLOv5s, enhancing steel surface defect detection with a 6.3% mAP increase and 15% FPS improvement by using C3DX and EIoU Loss.

Similarly, in other surface defect detection fields, deep learning techniques have shown significant advancements. For example, Brinatti Vazquez et al. [29] introduced SUPPOSe 3Dge, a method that achieves super-resolution surface defect detection in fluorescence microscopy, surpassing diffraction limits and enabling the high-precision reconstruction of fine structures. Additionally, Yun-Chao Tang et al. [30] proposed a novel crack width measurement method based on a dual-scale feature backbone, improving crack morphology estimation through an enhanced Zhang-Suen thinning algorithm and multi-step refinement process. Yang Yu et al. [31] proposed a hybrid framework for automated delamination detection in bridge decks using GPR, combining SSWT, CNN, transfer learning, and whale optimization, achieving over 94% accuracy. Lei Wang et al. [32] introduced an ultrasonic-based diagnosis method for concrete compressive damage amidst temperature variations, integrating continuous wavelet transform and deep CNNs to effectively identify damage despite environmental changes.

Despite these advancements, several challenges remain. The variety of defect shapes and sizes, the presence of multiple coexisting defects in a single image, and substantial background noise in the production environment make defect detection difficult. Moreover, the high-speed, batch-based nature of steel production requires real-time detection algorithms to ensure efficiency.

In response to these challenges, this study presents YOLOv10n-SFDC, an enhanced model based on YOLOv10. By optimizing the network architecture and incorporating a novel feature fusion module, YOLOv10n-SFDC significantly improves detection precision, robustness, and real-time performance, meeting the practical needs of industrial production. In particular, this research offers contributions in the following areas:

- Integrated into the backbone network, the DualConv module enhances the network’s ability to extract local features and optimizes the fusion of channel information. This improvement allows for more precise extractions of intricate features from the steel surface, especially when identifying smaller defects.
- The conventional feature fusion module is substituted with the SlimFusionCSP module. This module refines the combination of multi-scale features while maintaining computational efficiency, thus boosting the network’s ability to identify complex defects and accommodate multiple targets.
- To enhance the precision of border regression, the Shape-IoU loss function is introduced in this study, which prioritizes the correspondence between the predicted boundary shape and its actual dimension, significantly improving the model’s effectiveness in border regression tasks.
- This research achieves a reduction in model size by cutting down on unnecessary computations and minimizing the parameter count, thereby ensuring efficient and real-time operation in real-world industrial applications.

Experimental results on the NEU-DET dataset show that the YOLOv10n-SFDC model offers superior detection precision, speed, and robustness, especially in complex backgrounds and multi-defect scenarios. Compared to traditional methods and mainstream models, it achieves more accurate results while reducing computational overhead, making it a promising solution for industrial steel surface defect detection.

## 3. Overview of the YOLOv10

YOLOv10 [33] is a real-time, end-to-end object detection algorithm introduced in recent years. It builds upon the characteristics of the YOLO series while innovating and enhancing various aspects to further improve detection performance and efficiency. The primary advancements in YOLOv10 are centered on the post-processing strategy and model architecture design, with the goals of reducing computational overhead, increasing inference speed, and ensuring high-precision detection.

Among classic object detection algorithms, Mask R-CNN, RetinaNet, SSD, EfficientDet, CenterNet, and YOLO each offer unique strengths for different applications. Mask R-CNN [34] excels in segmentation quality but is computationally expensive, limiting real-time use. RetinaNet [35] improves accuracy for small objects with Focal Loss but increases computational and memory load. SSD [36], with its single-stage design, is suitable for real-time detection but struggles with small objects and high-resolution images. EfficientDet [37] optimizes computational efficiency and reduces memory usage, but performs poorly with complex backgrounds. CenterNet [38] balances speed and accuracy using keypoint detection, with moderate complexity and low memory demand, though it underperforms on small objects. YOLO is efficient and demands low memory, making it ideal for real-time detection in resource-constrained environments.

**Figure 1:**
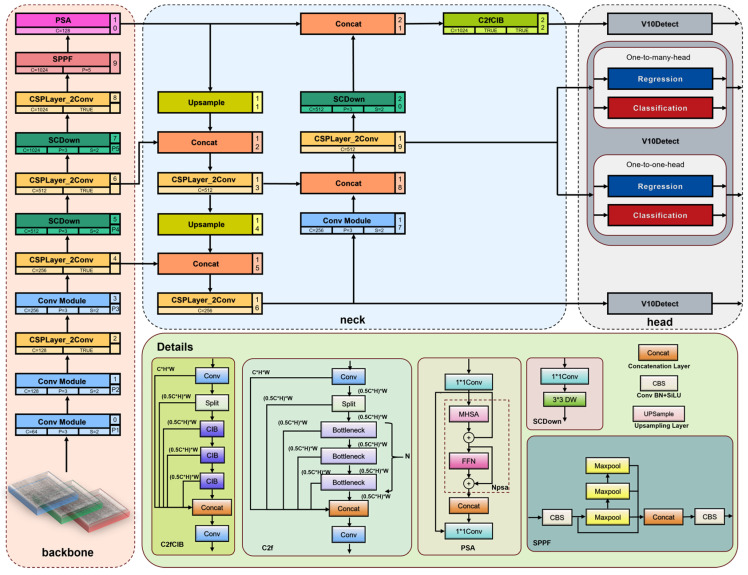
YOLOv10n architecture.

As shown in Figure 1, the architecture of the YOLOv10 network consists of four main components: the input, the backbone, the neck, and the head. During the input phase, data augmentation methods such as Mosaic [39] and Copy–Paste [40] were utilized to increase the variability of the training dataset, which helps to minimize overfitting and enhances the model’s ability to generalize in new situations. To ensure robustness against complex backgrounds and minimize noise interference, these augmentation techniques were carefully tuned. Specifically, the strength of these augmentations was dynamically adjusted to balance data diversity with noise suppression. Additionally, to counteract the possible negative impacts on model stability due to extensive data augmentation, these techniques were turned off during the last 10 epochs of the training process.

The backbone module is essential for feature extraction and includes components such as spatial channel decoupling downsampling (SCDown) for efficient feature reduction, the C2f component, Spatial Pyramid Pooling—Fast (SPPF) for improved feature representation, and the partial self-attention module (PSA) to refine feature maps by emphasizing relevant information. SCDown optimizes spatial and channel data processing, enhancing the model’s ability to extract features across different scales. SPPF broadens the receptive field, benefiting large target detection, while PSA improves small target detection by refining feature relationships with local self-attention.

In the neck module, YOLOv10 incorporates the Path Aggregation Feature Pyramid Network (PAFPN) [41], merging Feature Pyramid Network (FPN) and Path Aggregation Network (PANet) advantages for efficient multi-scale feature fusion, improving detection across varying target sizes.

In the head module, YOLOv10 uses a decoupled head structure with the C2fCIB module, an enhancement of YOLOv8’s C2f [42] and inspired by ELAN [43]. C2fCIB aggregates multi-level features, boosting small target detection and improving the receptive field and feature expression.

YOLOv10 reduces computational overhead with a lightweight classification head, eliminating the previous target branch and focusing on independent classification and regression branches. This improves precision and detection stability. Compared to YOLOv9-C [44], YOLOv10 reduces inference latency by 46% and parameters by 25%, enhancing efficiency and resource utilization. Additionally, it introduces a refined feature fusion strategy, improving stability and precision in various scenarios.

Indeed, YOLOv11 [45] and YOLOv12 are the most recently updated models, offering higher accuracy and optimized multi-scale feature fusion capabilities. YOLOv11 improves speed and accuracy, while YOLOv12 enhances small object detection. However, these updated models are not suitable for steel defect detection. YOLOv11 struggles with small object detection in complex backgrounds, and although YOLOv12 improves small object detection, its deeper network is prone to overfitting, making it difficult to accurately capture the fine details of steel defects. In contrast, YOLOv10 is more appropriate and mature for steel defect detection tasks.

## 4. YOLOv10n-SFDCs

**Figure 2:**
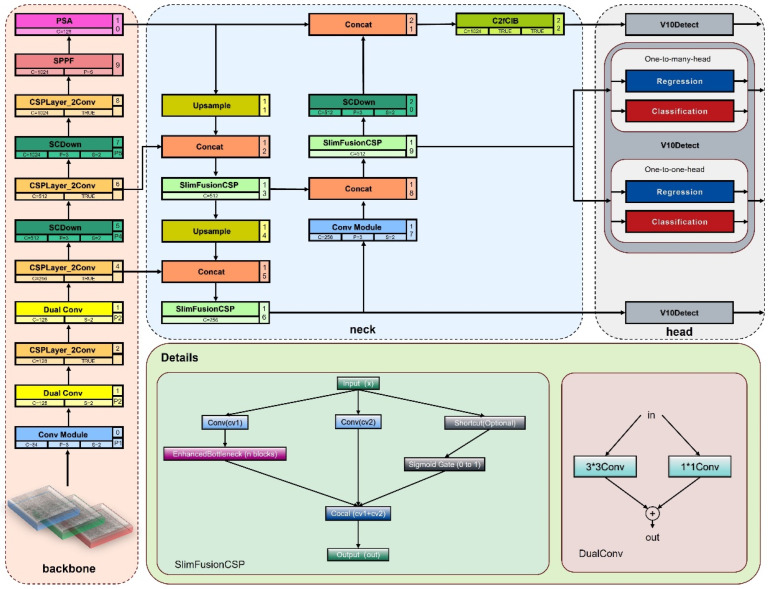
YOLOv10n-SFDC Architecture.

To enhance the suitability of YOLOv10 for identifying steel defects, the original YOLOV10N model was improved and optimized. The revised network structure is illustrated in Figure 2. First, the standard Conv module in the backbone network was replaced with the DualConv module to bolster the model’s ability to extract local features and integrate channel information. Next, in the head, the SlimFusionCSP module substituted the traditional C2f module, which reduced computational overhead by fusing features of varying scales, thereby significantly improving both inference speed and the model’s lightweight nature. Ultimately, the Shape-IoU was utilized in place of the CIoU loss function, focusing more on the shape and size of the bounding box to improve the accuracy of boundary regression. These key improvements have led to significant enhancements in the model’s precision in detecting defects, particularly when addressing defects of diverse sizes and shapes.

### 4.1. Loss Function Improvements

In the YOLOv10 model, the loss function plays a crucial role in positional regression, primarily aimed at optimizing the prediction precision of the bounding box. However, the traditional CIoU loss function [46] presents certain limitations when addressing complex shapes and multi-scale targets in steel defect detection tasks. Notably, when the shape and scale of the target differ significantly, the CIoU’s consideration of shape and scale in bounding box regression is insufficient, potentially resulting in substantial regression errors and hindering further improvements in model performance. To address these issues, the Shape-IoU [47] loss function (Equation (1)) has been introduced as an optimization scheme. Building on the traditional IoU, Shape-IoU enhances the robustness of bounding box regression by taking into account both the shape and scale of the bounding box. Specifically, by incorporating shape distance and shape constraint terms, Shape-IoU allows for a more comprehensive evaluation of the degree of matching between the predicted box and the actual box.

(1) $$L_{Shape-IoU}=1-IoU+{distance}_{shape}+0.5\cdot \Omega_{shape}$$

The IoU represents the ratio of overlap between the predicted bounding box and the ground truth box. The ${distance}_{shape}$ denotes a distance metric that accounts for both shape and scale characteristics, while $\Omega_{shape}$ serves as a shape constraint term designed to further balance the discrepancies in shape between bounding boxes. These enhancements enable Shape-IoU to measure more accurately the deviation between predicted and true bounding boxes, particularly in complex backgrounds. The key to Shape-IoU lies in its modeling of shape and scale characteristics: ${distance}_{shape}$ maps shape differences into a distance loss using a weighted approach, thereby enhancing the model’s localization precision for asymmetric targets, whereas $\Omega_{shape}$ quantifies the deviation in shape factors, such as aspect ratios, which further mitigates the misalignment of bounding box shapes.

Compared to CIoU, Shape-IoU demonstrates superior performance when handling overlapping defects or defects with irregular shapes. CIoU focuses primarily on the overlap area and center point distance, which can lead to regression errors in scenarios where bounding boxes overlap but have distinct shapes or aspect ratios. In such cases, Shape-IoU provides a more nuanced evaluation by incorporating shape distance and constraint terms. This enables the better differentiation of intersecting bounding boxes and improves localization precision for irregular targets. These advantages are particularly evident in steel defect detection tasks involving highly variable or overlapping defects, where Shape-IoU significantly reduces misalignment and enhances robustness against geometric complexities.

### 4.2. Dual_Conv Module

In the YOLOv10 architecture, the Conv module functions as the essential element for extracting features, usually capturing image details via conventional convolution processes. However, in the context of steel defect detection, the traditional Conv module exhibits certain limitations when confronted with complex backgrounds and multi-scale defects. This is particularly evident when addressing subtle defects and intricate textures, as the Conv module struggles to balance the consideration of both local details and global information, ultimately leading to constrained model performance. To address these challenges, the DualConv [48] module was developed. DualConv integrates 3 × 3 groups of convolutions with 1 × 1 point convolutions, thereby enhancing the capability for local feature extraction and channel information fusion simultaneously. By executing both convolution operations at the same level, DualConv efficiently extracts detailed image features while improving attention to critical areas within the global context.

**Figure 3:**
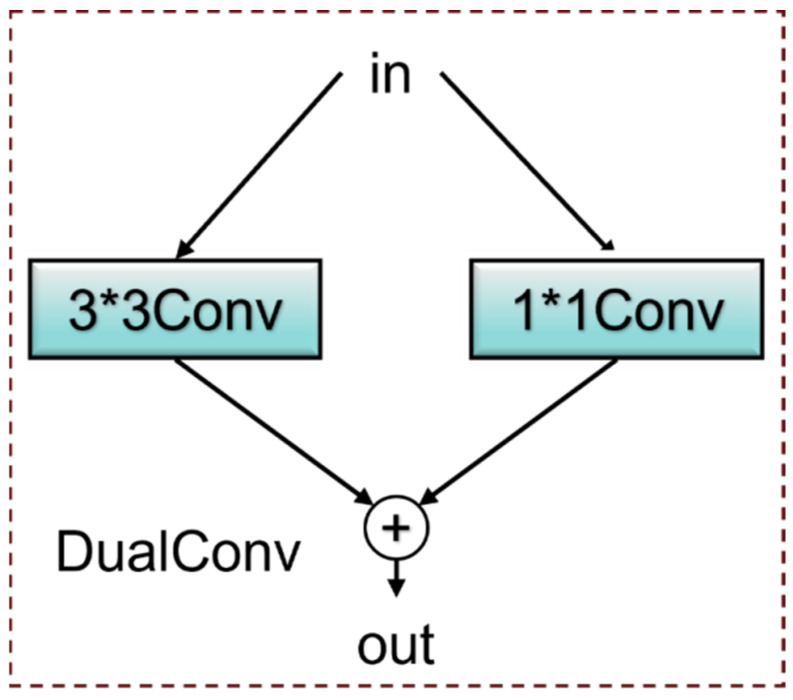
DualConv module structure.

Specifically, the 3 × 3 convolution focuses on capturing local spatial features critical for small defect detection, such as edges and textures, while the 1 × 1 convolution ensures the integration of cross-channel information, reducing the loss of contextual relevance. This synergy allows the module to effectively highlight subtle defect patterns, particularly in noisy or complex steel surfaces, where traditional convolution methods often fail to achieve sufficient sensitivity. The structure is illustrated in Figure 3, with the specific output of the DualConv presented in Equation (2).

(2) $$y=DualConv\left(x\right)=W_{3\times 3}*x+W_{1\times 1}*x$$

In this scenario, let x represent the input feature map, and let $W_{3\times 3}$ and $W_{1\times 1}$ indicate the convolution kernels sized 3 × 3 and 1 × 1, respectively. The convolution operation is denoted by the symbol *, while the output feature map is represented by y. The 3 × 3 convolution is utilized to capture local spatial features, whereas the 1 × 1 convolution functions to integrate information across different channels. This synergy allows the DualConv module to extract spatial and channel features simultaneously, thus improving the understanding of detail and overall information.

Unlike traditional convolution operations, DualConv not only enhances local feature extraction but also effectively captures the relationships between channels through 1 × 1 convolution. This design is particularly important in steel defect detection tasks, where defects are often partial and small, and the fusion of information between channels aids in improving the model’s adaptability to complex scenarios. Consequently, DualConv demonstrates greater robustness and precision when processing images with intricate backgrounds and multi-scale features.

### 4.3. SlimFusionCSP Module

#### 4.3.1. SlimFusionCSP Module Structure

**Figure 4:**
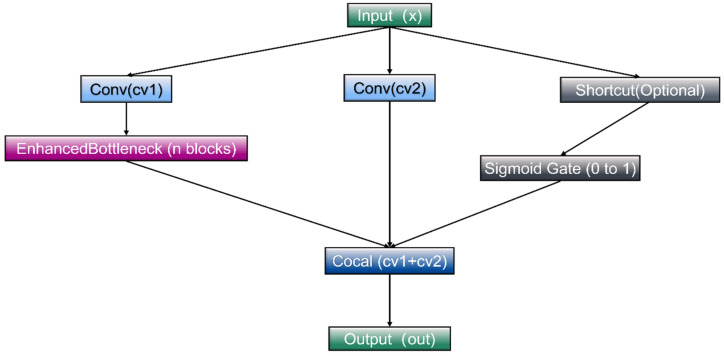
SlimFusionCSP module structure.

In the YOLOv10 architecture, the head’s C2f module is essential as it integrates and processes features obtained from the backbone network for the tasks of classification and location prediction. However, when applied to the detection of steel defects, the C2f module exhibits certain limitations. Specifically, the dimensions and shapes of imperfections on steel surfaces can vary significantly, while the background often contains irregular textures and noise, which increases the likelihood of both false positives and missed detections by the model. To address these challenges, the SlimFusionCSP module has been introduced as a replacement for the C2f module, resulting in a notable enhancement in the model’s effectiveness. The SlimFusionCSP module improves upon the conventional CSPNet [49] architecture, with the aim of enhancing feature fusion capabilities and overall learning efficiency in object detection tasks, as illustrated in Figure 4.

#### 4.3.2. Details of the SlimFusionCSP Module

The SlimFusionCSP module comprises two primary branches: the feature extraction path and the residual path. Initially, the input feature map is segmented into channels through two 1 × 1 convolution operations, resulting in the generation of two distinct feature maps. These maps are utilized for deep feature extraction and direct feature fusion, respectively. This design enhances the preservation of the original information while minimizing information loss. Specifically, the input feature map F produces feature maps $F_{1}$ and $F_{2}$ via the two convolution operations, as illustrated in Equations (3) and (4).

(3) $$F_{1}=Conv\left(F,W_{cv1},b_{cv1}\right)$$

(4) $$F_{2}=Conv\left(F,W_{cv2},b_{cv2}\right)$$

**Figure 5:**
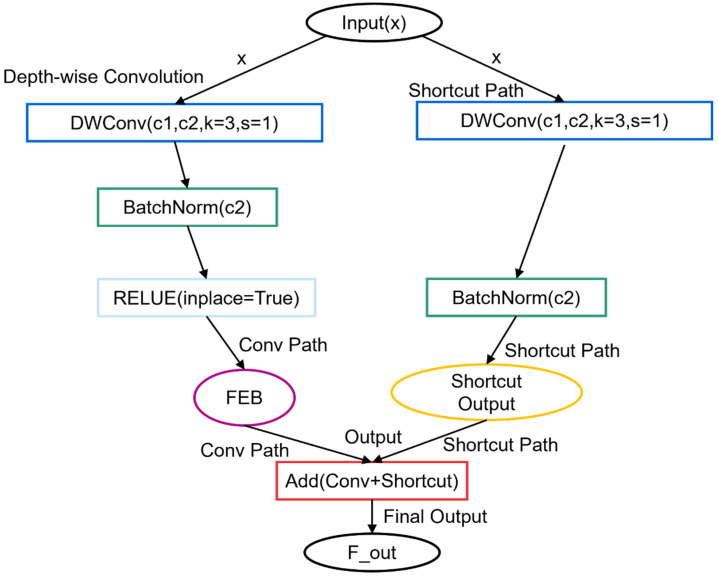
Structure of the EnhancedBottleneck Module.

$W_{cv1}$ and $W_{cv2}$ are convolution kernels, while $b_{cv1}$ and $b_{cv2}$ represent bias terms. $F_{1}$ and $F_{2}$ are feature maps generated through convolution operations. Subsequently, the feature map processed through the $F_{1}$ branch undergoes sequential processing via multiple EnhancedBottleneck modules. These modules employ deep separable convolutions and residual connections [50] to facilitate efficient feature information transfer while minimizing computational costs. The EnhancedBottleneck module in SlimFusionCSP is a structural enhancement for convolutional neural networks, building upon traditional bottleneck architectures (such as the Bottleneck in ResNet). This enhancement primarily incorporates deep separable convolutions, residual connections, and optimized activation functions to elevate feature extraction efficiency and overall model performance, as illustrated in Figure 5.

The core structure of the module consists of two main components: one for extracting depth features through depth separable convolution and the other for preserving input features via residual connections. Initially, the input feature map undergoes depth separable convolution, which significantly decreases computational requirements by decoupling spatial convolution from channel convolution. In traditional convolution, a convolution kernel is needed for each input channel, whereas depth separable convolution employs a distinct convolution kernel for each input channel, thereby greatly reducing computational complexity. Following the depth convolution operation, the output is subjected to batch normalization [51], which aids in minimizing internal covariate shift and accelerating the training process. Subsequently, a nonlinear transformation is applied through the ReLU activation function to enhance the network’s expressive capability.

#### 4.3.3. Residual Connection and Gating Mechanisms

To enable the network to disseminate information efficiently, the EnhancedBottleneck introduces residual connections. The fundamental concept of a residual connection is to add the input feature map to the result of the convolution operation, producing the final output. This design helps alleviate the vanishing gradient problem, allowing information to be transmitted more effectively at deeper layers, thus facilitating the network’s training and optimization. The EnhancedBottleneck module processes the input feature map through depth separable convolutions and combines it with the feature map processed by another depth separable convolution and batch normalization, enhancing the model’s learning capability and performance while maintaining high computational efficiency.

The particular advantage of depth separable convolutions in steel defect detection lies in their significant reduction in computational cost and parameter count, while preserving important spatial and local features. In steel defect detection tasks, subtle defect features are often difficult to capture. Depth separable convolutions split the convolution operation into two independent steps (depthwise convolution and pointwise convolution), not only improving computational efficiency but also increasing the model’s sensitivity to detailed features when processing high-dimensional data. This is especially advantageous for detecting small defects on the steel surface, particularly in low-contrast or noisy conditions.

In this way, the EnhancedBottleneck module not only improves the network’s efficiency but also optimizes information extraction and detail processing for the steel defect detection task, enabling the better capture and recognition of small steel defects. Let the output of the EnhancedBottleneck module be denoted as $F_{EB}$ , as shown in Equation (5).

$DWConv\left(F_{1}\right)$ denotes a deeply separable convolution operation, while Residual refers to a residual concatenation operation. The feature map $F_{2}$ , produced by the branch of the feature map $F_{EB}$ and $F_{2}$ of EnhancedBottleneck, will be concatenated along the channel dimension. This channel-by-channel connection operation facilitates feature fusion, allowing for the retention of more feature information and the effective integration of deep and shallow features. The spliced feature diagram $F_{concat}$ is illustrated in Equation (6).

(5) $$F_{EB}=Residual(DWConv\left(F_{1}\right),\;F_{1})$$

(6) $$F_{concat}=Concat(F_{2},\;F_{EB})$$

In this step, the channels of the two feature maps are merged to create a more comprehensive fusion feature map, denoted as $F_{fusion}$ . Subsequently, the concatenated feature map $F_{concat}$ undergoes further feature fusion via a 1 × 1 convolution, $W_{cv3}$ , resulting in a preliminary output feature map, $F_{fusion}$ (as described in Equation (7)). This process also adjusts the number of channels to the target output number, ensuring compatibility with the downstream module.

(7) $$F_{fusion}=Conv(F_{concat},W_{cv3},\;b_{cv3})$$

To further optimize feature fusion, the SlimFusionCSP module introduces a gating mechanism [52]. This mechanism dynamically adjusts the fusion priority of the feature map, enabling the model to selectively transmit key information to the subsequent layer based on task requirements. This approach prevents the transmission of redundant information and mitigates the impact of irrelevant features on the final prediction results. Consequently, the model can effectively suppress noise in complex backgrounds, enhancing its focus on steel defect areas and thereby improving the precision of steel defect detection. The gating process is facilitated by a learnable gating variable. This variable is transformed into a value between 0 and 1 using the sigmoid function, which is then weighted with the residual concatenation. The final output feature plot $F_{out}$ is expressed by the formula shown in Equation (8).

(8) $$F_{out}=F_{fusion}+\sigma (gate)\cdot F_{shortcut}(x)$$

In this context, σ denotes the sigmoid function, gate refers to the trainable gating variable, and $F_{shortcut}(x)$ represents the shortcut connection of the input. The SlimFusionCSP module enhances feature extraction by incorporating residual connections and gating mechanisms. This design allows it to preserve the integrity of original information while dynamically adjusting the weighting of various features based on the particular demands of the task at hand. Through this approach, the module ensures that critical information is retained and appropriately emphasized for optimal performance.The incorporation of the gating mechanism significantly enhances the model’s focus on crucial information, mitigates the influence of irrelevant features, and ensures the stability of the gradient, thereby preventing issues related to gradient vanishing or explosion. By integrating deep separable convolution, residual connections, and a gating mechanism, the SlimFusion CSP module significantly enhances the capacity and robustness of feature extraction while preserving the lightweight nature of the CSP structure. This module improves the performance of YOLOv10 in intricate visual tasks, particularly in multi-scale feature fusion and defect recognition within complex backgrounds, demonstrating superior feature expression capabilities and stability.

The SlimFusionCSP module incorporates a gating mechanism that dynamically adjusts the influence of the shortcut connection based on task-specific requirements. The gate is initialized as a learnable parameter and transformed into a value between 0 and 1 using a sigmoid function. During the forward pass, this gate controls the contribution of the shortcut connection to the output. Specifically, higher gate values indicate greater reliance on the shortcut connection for feature fusion, while lower values diminish its influence. This implementation enables the quantitative analysis of the gating mechanism. By monitoring the gate variable during training, it is possible to observe how the model dynamically adjusts the weighting of the shortcut connection. For example, the gate values can reveal the extent to which the model prioritizes feature reuse versus new feature extraction under different training conditions.

The SlimFusionCSP module achieves an effective balance between feature fusion efficiency and computational demands through the combined use of depthwise separable convolutions [53], residual connections, and the gating mechanism. Depthwise separable convolutions significantly reduce computational cost while preserving spatial and local features, enhancing sensitivity to subtle defect details. Residual connections ensure efficient information transfer and stabilize gradient flow, allowing the model to retain critical feature information. Meanwhile, the gating mechanism selectively emphasizes key features and suppresses irrelevant ones, adapting dynamically to complex defect scenarios. Together, these components enable the SlimFusionCSP module to optimize feature fusion while maintaining a lightweight and computationally efficient design, ensuring robust performance even in challenging detection tasks.

## 5. Experiment

### 5.1. Dataset Details

This study utilizes a dataset provided by Northeastern University, known as NEU-DET, which is specifically designed to document defects on steel surfaces. The dataset encompasses six typical categories of steel surface defects: cracks (Cr), inclusions (In), plaques (Pa), scratches (Sc), oxidized rolled skin (Rs), and pitting surfaces (Ps). In total, the dataset includes 300 images for each defect type, with all images sized at 200 × 200 pixels. The images were randomly allocated to a training set and a validation set at a 9:1 ratio, resulting in a training set comprising 1620 images and a validation set containing 180 images.

To verify the robustness and generalization ability of the model, experiments were conducted using the surface defect dataset GC10-DET, which was collected from real industrial scenarios. The GC10-DET dataset encompasses ten distinct types of surface defects, with the following labels: 329 punching holes (Pu), 513 welding lines (Wl), 265 crescent gaps (Cg), 354 water spots (Ws), 569 oil spots (Os), 884 silk spots (Ss), 347 inclusions (In), 85 rolling pits (Rp), 74 creases (Cr), and 143 waist creases (Wf). All defects are sourced from the surface of steel plates. The dataset comprises 3563 grayscale images, each with a resolution of 2048 × 1000 pixels. The dataset was divided into a training set and a validation set in an 80:20 ratio. This division was chosen due to the high quality and clarity of the images, as well as the relatively large dataset size. With clear and detailed defect features, the larger dataset allows for more effective training and validation, ensuring that the model learns to generalize well across different defect types while minimizing overfitting.

### 5.2. Experimental Environment and Evaluation Indicators

The study’s experimental setup was conducted on a Windows 10 OS featuring 128 GB of RAM. The machine was powered by an Intel(R) Xeon(R) Silver 4214R processor, which operated at 2.40 GHz, alongside an NVIDIA GeForce RTX 3090 graphics card. The software frameworks used included Python version 3.9, PyTorch 1.11.0, and CUDA 11.3. During the training phase, the model underwent 300 epochs, utilized a batch size of 32, and handled input images with a resolution of 640 × 640.

In this research, we utilized an extensive array of evaluation metrics to evaluate the performance of the model. The metrics used encompass precision, recall, a mean Average Precision at an Intersection over Union (IoU) threshold of 0.5 (mAP50), as well as computational efficiency, expressed in billions of floating-point operations per second (GFLOPs). These metrics offer valuable information regarding both the accuracy of the model and its computational requirements, along with the complete count of model parameters (Params). Collectively, these metrics provide a thorough evaluation of the model’s performance and efficiency. Precision reflects the ratio of true positive samples within the predicted true class samples, while recall signifies the proportion of true samples that the model accurately identifies as positive from the total actual true class samples. Furthermore, mAP signifies the average precision, whereas mAP50 indicates the setting of the IoU threshold at 0.5 during the mAP calculation. Within this framework, a detection is considered accurate when the Intersection over Union (IoU) with the actual target surpasses 0.5, which is a widely used threshold for dependable object detection assessments. GFLOPs serves as a metric for the model’s computational efficiency, revealing the number of billions of floating-point operations executed per second. In contrast, Params represents the complexity and scale of the model by indicating the total parameter count, thereby shedding light on its architecture and learning capabilities. The formulas for calculating precision, recall, and mAP are delineated in Equations (9)–(11).

(9) $$Precision=\frac{TP}{TP+FP}$$

(10) $$Recall=\frac{TP}{TP+FN}$$

(11) $$mAP=\frac{1}{c}\sum_{i=1}^{c}AP_{i}$$

### 5.3. Analysis of Experimental Results

As shown in Table 1, the improved model demonstrates significant performance improvements across various categories of defect detection tasks, especially in the detection of more complex objects. Notably, the “pitted surface” category exhibits the highest performance, with precision reaching 0.904, recall at 1.0, and a nearly perfect mAP50 of 0.995, highlighting the model’s exceptional capability for defect detection. In contrast to the baseline, which showed strong performance in this category, the improved model further optimizes the detection accuracy.

For the “rolled in scale” category, the mAP50 value increases to 0.884, showing a significant enhancement in precision and recall compared to the baseline method’s performance, which struggled in detecting such defects. The “scratches” category shows a slight decrease in mAP50 from the baseline, from 0.852 to 0.822, yet the overall performance still remains robust compared to the baseline model.

In the more challenging “crazing” category, the improved model’s precision remains lower at 0.514, but recall has improved significantly to 0.75, and the mAP50 increases to 0.604, indicating progress in reducing false negatives. This shows that while challenges remain, the model is becoming more reliable overall.

The “inclusion” category also sees a noticeable improvement in mAP50, reaching 0.861, compared to the baseline of 0.752. The “patches” category reaches 0.966, showing an impressive enhancement, with a small increase of 0.052 over the baseline’s mAP50 of 0.914.

Overall, the enhanced model strikes a good balance between precision, recall, and lightweight design, making it well suited for multi-class defect detection tasks. The comparison against the baseline reveals notable improvements, especially in precision and recall for more complex defect types.

In order to rigorously evaluate and validate the effectiveness of the proposed enhancements, this study carried out four ablation experiments. These experiments were conducted under uniform environmental and training parameters to ensure comparability. Both the experimental and control groups underwent training and testing phases, with all corresponding outcomes carefully documented. The detailed results of the ablation experiments are presented in Table 2.

In the task of detecting steel surface defects, YOLOv10 was optimized by incrementally introducing improvement modules and assessing their impact on performance. Changes in mAP50 values, GFLOPs, and parameter counts were recorded throughout. The unmodified YOLOv10 model achieved an mAP50 of 0.792, GFLOPs of 8.2, and a parameter count of 2.69 M, serving as the baseline. Integrating the Shape-IoU module improved bounding box regression, increasing the mAP50 to 0.816, with GFLOPs and parameters rising to 8.88 and 2.82 M, respectively. Adding the DualConv module further enhanced the mAP50 to 0.830, with GFLOPs and parameters recorded at 8.48 and 2.77 M, improving local feature extraction and channel information fusion. Finally, incorporating the SlimFusionCSP module achieved an mAP50 of 0.855, while reducing GFLOPs to 8.10 and parameters to 2.67 M, demonstrating improved feature fusion efficiency with lower computational overhead.

These experiments highlight the individual contributions of Shape-IoU, DualConv, and SlimFusionCSP to detection performance. While the modules were introduced sequentially to isolate their impacts, combining these enhancements earlier in training could potentially yield greater gains by leveraging their synergistic effects. Shape-IoU’s robust bounding box regression, combined with DualConv’s spatial and channel feature extraction, could enhance defect localization and fine-grained detail capture from the outset. The SlimFusionCSP module’s lightweight design and efficient feature fusion would further amplify these benefits, especially for multi-scale and irregular targets. Early integration might also accelerate convergence by enabling more effective feature representation and optimization.

### 5.4. Comparison Experiment

**Figure 6:**
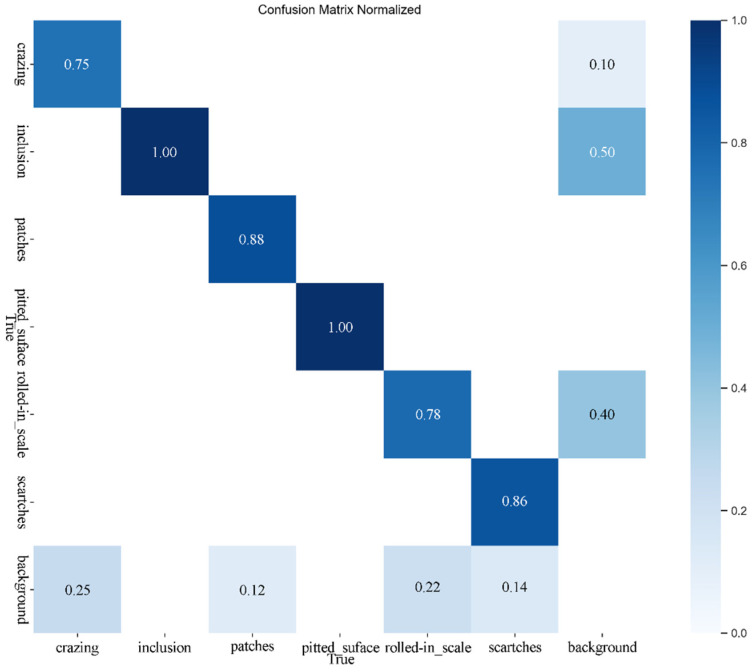
Confusion matrix of our model on the NEU-DET dataset.

**Figure 7:**
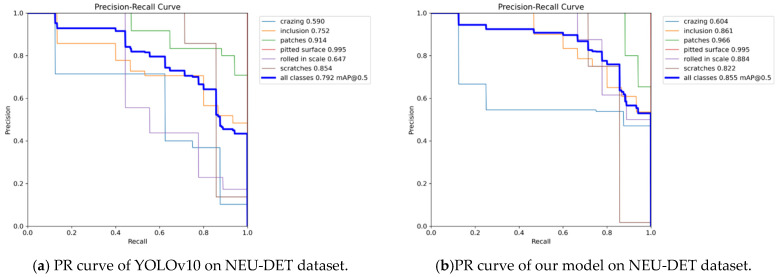
PR curves of YOLOv10 and our proposed model on the NEU-DET dataset.

**Figure 8:**
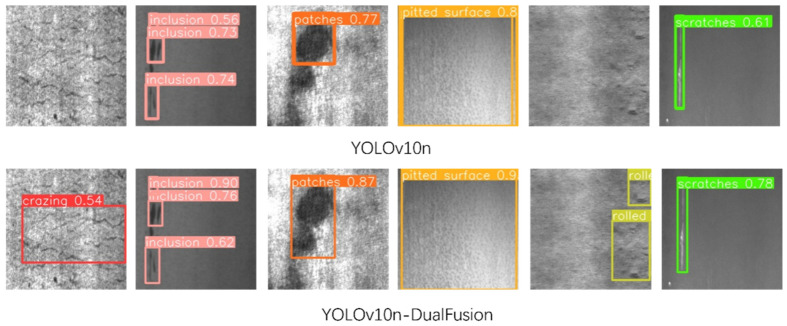
Comparison of results on the NEU-DET dataset between YOLOv10 and ours.

**Figure 9:**
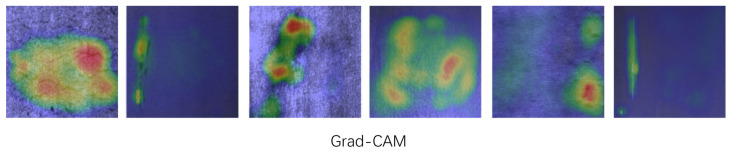
Grad-CAM visualization on the NEU-DET dataset using our model.

As shown in Figure 6, the confusion matrix for the upgraded model is depicted, with the horizontal axis representing the actual values and the vertical axis representing the predicted values. This matrix serves as a visual summary, reflecting the model’s performance by comparing real outcomes to predictions. The graph indicates that most predictions are consistent with the actual values, demonstrating a robust predictive capability of the model. Under the same experimental conditions, the detection performance of the revised YOLOv10n-SFDC exhibits notable differences from the original YOLOv10 in various aspects. Figure 7 displays the precision–recall curves along with the respective mAP50 values for both the YOLOv10 model and its modified version, YOLOv10n-SFDC. This illustration offers a comparative view of the performance metrics for the two models. Moreover, Figure 8 presents the prediction outcomes before and after the enhancements applied to YOLOv10. The scaled graph indicates that the upgraded model not only effectively recognized categories such as “crazing” and “rolled in scale”, which were overlooked by the original model, but also markedly improved the detection rates for the “inclusion” category from 0.56 to 0.90, the “patches” category from 0.77 to 0.87, the “pitted” category, the surface category from 0.80 to 0.90, and the scratches category from 0.61 to 0.78. In contrast, the proposed Shape-IoU loss function significantly reduced regression errors by considering both shape and scale, leading to more precise bounding boxes and improved detection outcomes. Figure 9 presents the Grad-CAM heatmaps for the predictions made by the upgraded YOLOv10n-SFDC model on the NEU-DET dataset. The heatmaps visually highlight areas where the model has focused its attention, with regions of higher importance represented by warmer colors (yellow to red). These visualizations demonstrate that the enhanced model not only effectively identifies defect categories that the original model missed, such as “crazing” and “rolled in scale”, but also provides greater clarity and precision in defect localization, further validating the improvements in detection performance.

The enhanced YOLOv10n-SFDC model increased the overall mAP from 79.2% to 85.5%, representing a rise of 6.3 percentage points. Notably, in the ‘rolled in scale’ category, the original YOLOv10 achieved an mAP value of only 0.647, whereas the YOLOv10n-SFDC improved this to 0.884, indicating a significant performance enhancement for this challenging category. Additionally, other categories such as ‘inclusion’ and ‘patches’ also saw mAP increases of 0.103 and 0.052, respectively. This suggests that the improved algorithm exhibits greater stability and precision when addressing complex defects and difficult-to-detect targets. In other categories, the YOLOv10n-SFDC continues to perform well. For instance, in the ‘crazing’ category, the mAP of the enhanced model rose from 0.590 to 0.604; in the ‘inclusion’ category, it increased from 0.758 to 0.861; in the ‘patches’ category, from 0.914 to 0.966; and in the ‘pitted_surface’ category, both YOLOv10n and YOLOv10n-SFDC achieved mAP values of 0.995. However, in the ‘scratches’ category, the mAP for YOLOv10n-SFDC was recorded at 0.822, which is slightly lower than YOLOv10n’s 0.854. Nevertheless, this minor decrease does not significantly impact the overall performance improvement of the model. Furthermore, the improved YOLOv10n-SFDC model comprises only 2.67 M parameters, which is notably lower than the 3.00 M parameters of YOLOv10n, underscoring its advantages in lightweight design. When compared with DETR, a transformer-based model designed for object detection, YOLOv10n-SFDC exhibits distinct advantages in both computational efficiency and detection precision. While DETR achieved a respectable mAP of 0.666 with 36.7 M parameters, its heavy reliance on transformer layers leads to substantial computational overhead, which can be a limitation for real-time and resource-constrained applications. In contrast, YOLOv10n-SFDC not only outperforms DETR in mAP (0.855 vs. 0.666), but also achieves this with over 13× fewer parameters. These results underscore the effectiveness of YOLOv10n-SFDC’s architecture in balancing lightweight design and high precision, making it a more suitable choice for industrial applications requiring rapid and efficient defect detection.

Although additional modules were embedded into YOLOv10n to create YOLOv10n-SFDC, the model’s parameter size decreased, demonstrating the efficiency of the proposed structural optimizations. This reduction in parameter size can be attributed to the introduction of the SlimFusionCSP and dual convolution modules, which enhance feature extraction and fusion while minimizing computational overhead. To further emphasize the lightweight design, Table 3 provides a comparative analysis of parameter sizes across models, highlighting that YOLOv10n-SFDC achieves superior detection performance with significantly fewer parameters compared to traditional models like SSD (24.1 M) and DETR (36.7 M). These results validate the proposed method’s ability to balance lightweight design and high detection precision.

### 5.5. Model Performance Verification(on the GC10-DET Dataset)

In this study, experiments were conducted on the surface defect dataset GC10-DET, collected from real industrial scenarios, to evaluate the performance of the model. The experimental results are summarized in Table 4 and Table 5. The YOLOv10n-SFDC model, trained with the same parameters for both the NEU-DET and GC10-DET datasets, demonstrated strong performance on the GC10-DET dataset, achieving a precision of 63.7%, recall rate of 61.7%, and mAP50 score of 65.6%, showing its effective generalization across datasets. Although the GC10-DET dataset is derived from a real industrial environment, leading to slightly lower detection precision compared to other laboratory datasets, it offers valuable insights for practical applications.

Table 4 compares the results of the NEU-DET and GC10-DET datasets. The YOLOv10n-SFDC model outperforms YOLOv10 in most cases. Specifically, for the NEU-DET dataset, the model achieved a precision of 71.8%, recall rate of 85.4%, and mAP50 score of 85.5%, compared to the YOLOv10 baseline of 79.2%. For the GC10-DET dataset, the precision was 63.7%, recall rate was 61.7%, and mAP50 was 65.6%, with YOLOv10 achieving a slightly lower mAP50 of 64.4%.

Notably, the model excelled in detecting defects in the punching category, achieving a precision of 0.802, recall rate of 0.963, and mAP50 score of 0.96. This highlights the model’s effectiveness in accurately detecting and minimizing missed detections in this category. The performance in the crescent-shaped gap and weld categories was also commendable, with precision scores of 0.78 and 0.768, recall rates of 0.959 and 0.907, and mAP50 scores of 0.937 and 0.912, respectively. These results underscore the model’s enhanced capability to detect these specific types of defects, demonstrating its robustness and reliability.

For the water spot category, the model achieved a precision of 0.687, recall rate of 0.807, and mAP50 score of 0.834, indicating strong detection performance. While slight deficiencies were observed in precision and recall for other categories such as oil spots, silk spots, and creases, the overall performance remained satisfactory without any major issues. Furthermore, when tested on a separate dataset containing ten categories, the model accurately identified the crack category and its location, distinguishing it clearly from the background. This further demonstrates the model’s strong robustness and generalization capabilities.

### 5.6. Model Generalization Verification (On the Welding Defect Dataset)

In order to verify the generalization ability of the model proposed in this study, a dataset specifically designed for detecting defects in welding surfaces is utilized. The dataset includes three distinct categories: bad weld, good weld, and defect. The images in this dataset are sourced from various image collections and datasets and are formatted in the YOLO annotation format. The dataset was randomly split into a training set and a validation set at an 8:2 ratio, with the majority of the images allocated to the training set and the remaining to the validation set.

As shown in Table 6,although the model shows significant improvement over the baseline on the NEU steel defect dataset, its performance gain on the welding defect dataset is limited, indicating that its generalization ability needs further enhancement. Specifically, the precision and recall for the defect category are low, and the model’s advantage over the baseline is not apparent. This suggests that the model’s generalization is not yet ideal and requires further refinement, particularly for diverse real-world applications. Future work could explore more advanced model architectures and data augmentation strategies to improve performance across various datasets and scenarios.

## 6. Discussion

In this study, we present an enhanced version of the YOLOv10 model, named YOLOv10n-SFDC, specifically designed for defect detection on steel surfaces. The model integrates several key innovations, including the DualConv module, the SlimFusionCSP module, and the Shape-IoU loss function. These improvements collectively enhance the model’s precision and robustness while maintaining minimal computational costs. The DualConv module captures both local details and global context, critical for detecting small defects, while the SlimFusionCSP module optimizes feature fusion and accelerates inference. The Shape-IoU loss function places greater emphasis on the shape and scale of target bounding boxes, leading to more accurate bounding box regression.

The YOLOv10n-SFDC model demonstrates superior performance with a mean Average Precision (mAP) of 85.5%, outperforming previous models. Specifically, it surpasses the YOLOv8-based model proposed by Xuan Song et al. [56], which incorporates deformable convolutions and the BiFormer attention mechanism, achieving an mAP of 84.8%, and the STE-YOLO model introduced by Dongming Li et al. [57], which utilizes GhostConv and a Bottleneck Transformer self-attention convolution layer, achieving an mAP of 79.0%. Despite both models showing improvements of 6.9% and 13.1% over the original YOLOv8, respectively, they still lag behind YOLOv10n-SFDC in handling small, multi-scale defects, particularly in complex and cluttered environments. These advancements allow YOLOv10n-SFDC to outperform these previous models, demonstrating its superiority in diverse and challenging industrial defect detection scenarios.

## 7. Conclusions

Although the YOLOv10n-SFDC model has made significant advancements in steel surface defect detection, it still faces challenges, particularly in environments with complex backgrounds and the presence of multiple overlapping objects. Future research should focus on enhancing the model’s detection capabilities in such cluttered scenarios, with an emphasis on improving background noise suppression and strengthening its robustness. Additionally, as datasets become more diverse and larger, exploring data augmentation and self-supervised learning techniques to improve the model’s generalization will be crucial. The YOLOv10n-SFDC model also holds strong potential for defect detection across industries such as aerospace, automotive, and electronics. In the future, optimizing the model for real-time deployment in industrial-scale applications, particularly on edge devices with limited computational resources, will be vital for its successful implementation in practical settings.

## Figures and Tables

**Table 1:** NEU-DET Dataset Results.

Class	Precision	Recall	MAP50	MAP50 (yolov10n)	MAP50 Difference
crazing	0.514	0.75	0.604	0.590	0.014
inclusion	0.598	0.892	0.861	0.752	0.109
patches	0.95	0.882	0.966	0.914	0.052
pitted surface	0.904	1	0.995	0.995	0.000
rolled in scale	0.603	0.889	0.884	0.647	0.237
scratches	0.732	0.714	0.822	0.852	−0.030

**Table 2:** Results of ablation experiments on NEU-DET dataset.

Sequence	Shape-IoU	Dual	SlimFusionCSP	mAP50	GFLOPs	Params
1				0.792	8.1	3.00
2	√			0.816	8.88	2.82
3		√		0.815	8.60	2.87
4			√	0.806	7.90	2.91
5	√	√		0.830	8.48	2.77
6	√	√	√	0.855	8.10	2.67

**Table 3:** Comparison of detection performance of different models.

Types	SSD [54]	Fast RCNN [55]	DETR [56]	Yolov7	Yolov8n	Yolov10n	Ours
crazing	0.627	0.422	0.268	0.318	0.630	0.590	0.604
inclusion	0.756	0.780	0.660	0.623	0.657	0.758	0.861
patches	0.943	0.925	0.890	0.940	0.905	0.914	0.966
pitted surface	0.715	0.861	0.715	0.995	0.995	0.995	0.995
rolled in scale	0.656	0.655	0.570	0.620	0.698	0.647	0.884
scratches	0.825	0.970	0.905	0.735	0.831	0.854	0.822
mAP	0.754	0.767	0.666	0.715	0.786	0.792	0.855
Params (M)	24.1	137.3	36.7	37.2	3.20	3.00	2.67

**Table 4:** Comparison of NEU-DET and GC10-DET dataset results.

Dataset	Precision	Recall	mAP50	mAP50 (yolov10)
NEU-DET	0.718	0.854	0.855	0.792
GC10-DET	0.637	0.617	0.656	0.644

**Table 5:** GC10-DET dataset results.

Class	Precision	Recall	mAP50	mAP50 (yolov10)
Pu	0.802	0.963	0.96	0.957
Wl	0.768	0.907	0.912	0.904
Cg	0.78	0.959	0.937	0.926
Ws	0.687	0.807	0.834	0.863
Os	0.634	0.599	0.636	0.621
Ss	0.6	0.509	0.535	0.571
In	0.387	0.192	0.254	0.248
Rp	0.219	0.118	0.138	0.108
Cr	0.756	0.311	0.543	0.464
Wf	0.732	0.806	0.813	0.777

**Table 6:** Welding defect dataset results.

Class	Precision	Recall	mAP50	mAP50 (yolov10)
ALL	0.682	0.692	0.693	0.682
BAD	0.728	0.76	0.789	0.776
GOOD	0.738	0.818	0.826	0.82
DEFECT	0.583	0.497	0.463	0.449

## Data Availability

The datasets utilized in this research, namely NEU-DET, GC10-DET, and Welding Defect-Object Detection, are publicly accessible. You can obtain these datasets from the following links: http://faculty.neu.edu.cn/songkechen/zh_CN/zhym/263269/list/index.htm; accessed on 12 June 2024. https://github.com/lvxiaoming2019/GC10-DET-Metallic-Surface-Defect-Datasets; accessed on 20 October 2024. https://www.kaggle.com/datasets/sukmaadhiwijaya/welding-defect-object-detection/data. accessed on 10 January 2025.
